# Neuromodulator Dynamics Underlying Associative Learning in the Ventral Striatum's Olfactory Tubercle

**DOI:** 10.1002/advs.74973

**Published:** 2026-03-23

**Authors:** Maojun Hong, Liuting Zou, Yiqing Chen, Juan Guo, Zhixiang Xu, Tong Ma, Shuaishuai Hu, Rongfeng K. Hu

**Affiliations:** ^1^ Shanghai Pudong Hospital Institute for Translational Brain Research State Key Laboratory of Brain Function and Disorders MOE Frontiers Center for Brain Science Fudan University Shanghai China; ^2^ Department of Anesthesiology Zhongshan Hospital Fudan University Shanghai China; ^3^ State Key Laboratory of Brain Function and Disorders MOE Frontiers Center for Brain Science Fudan University Shanghai China; ^4^ Institute for Translational Brain Research State Key Laboratory of Brain Function and Disorders MOE Frontiers Center for Brain Science Fudan University Shanghai China; ^5^ Shanghai Mental Health Center School of Medicine Shanghai Jiao Tong University Shanghai China

**Keywords:** dopamine, learning, neuromodulator dynamics, olfactory tubercle, reversal, reward

## Abstract

The brain's neuromodulatory systems—particularly dopamine (DA), serotonin (5‐HT), acetylcholine (ACh), and norepinephrine (NE)—play a crucial role in reward processing and associative learning. The olfactory tubercle (OT), a region that overlaps with the olfactory cortex and ventral striatum, has been implicated in non‐olfactory functions such as learning and motivation. Moreover, it receives dense innervation from multiple neuromodulator‐producing brain regions. Yet, how neuromodulator dynamics in the OT encodes external rewards and shapes different forms of associative learning remains unclear. Using fiber photometry and genetically encoded sensors, we captured reward‐evoked release patterns of four neuromodulators in the OT and tracked their dynamics across distinct learning processes. We uncover sex‐specific, state‐dependent, and type‐selective response dynamics to external rewards. Moreover, OT neuromodulators encode reward learning and extinction in a sexually dimorphic and state‐dependent manner. Notably, unlike the nucleus accumbens (NAc), OT dopamine does not encode reward prediction error. Finally, these neuromodulators dynamically track cue discrimination and reversal learning. Together, we present a systematic framework mapping how OT neuromodulation encodes reward processing and associative learning. This advances our understanding of OT's roles in both healthy cognition and neuropsychiatric disorders, such as addiction and depression.

## Graphical abstract

Using fiber photometry and genetically encoded sensors, the dynamic release patterns of four neuromodulators in the olfactory tubercle (OT)—a brain region overlapping with the olfactory cortex and ventral striatum—were systematically monitored in response to external rewards and distinct reward‐associated learning processes. The findings reveal sex‐specific, state‐dependent, and type‐selective response dynamics.

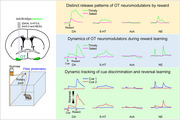

## 1 Introduction

Key neuromodulatory systems—dopamine (DA), serotonin (5‐HT), acetylcholine (ACh), and norepinephrine (NE)—orchestrate behavioral states and underlie synaptic plasticity during learning and decision‐making [1, 2, 3, 4]. Each system supports a unique set of processes: DA in reward and motivation, 5‐HT in mood and homeostasis, ACh in attention and memory, and NE in alertness and arousal [1, 2, 3, 4]. Until recently, advancements in genetically encoded sensors have enabled precise spatiotemporal measurement of individual neuromodulators in vivo, facilitating the investigation of their modulatory roles within specific neural circuits [1, 5]. Among these neuromodulators, striatal dopamine dynamics have been extensively studied for their roles in motivation, learning, and decision‐making [6, 7, 8, 9, 10, 11, 12, 13, 14, 15]. However, given the high interconnectivity of these neuromodulator systems [1, 5], a central question remains: what are the specific roles of distinct neuromodulators within the same brain region under identical conditions? Critically, recent studies have begun to explore these interactions to specific brain regions and functions, such as dorsal striatal DA‐ACh in reward‐based decision‐making [13, 15], ventral striatal DA‐5‐HT in reinforcement learning [16], and frontal ACh‐NE in competitive decision‐making [17]. While these studies have significantly advanced our understanding of neuromodulator dynamics, a more comprehensive investigation is essential. Potential differences in reward magnitude, biological sex, internal states, behavioral contexts, and the neuromodulator systems themselves must be addressed to achieve a complete understanding of how these mechanisms shape circuit function and behavior [1, 11, 18, 19, 20, 21, 22].

The ventral striatum (VS), a key reward region in the basal forebrain, is primarily composed of the nucleus accumbens (NAc) and the olfactory tubercle (OT) [23, 24, 25]. In contrast to the extensively studied NAc, the OT has received far less attention. This unique region bridges the olfactory cortex and ventral striatum and is now known to be critical for various non‐olfactory processes—including reward processing, associative and reversal learning, and motivated behaviors [23, 24, 25, 26, 27, 28, 29, 30, 31, 32]. Previous anatomical and immunostaining studies have established that the OT receives dense neuromodulatory innervation and expresses a rich diversity of neuromodulator receptors [24, 25, 26, 33, 34, 35]. However, the in vivo dynamics of these neuromodulators during reward processing and associative learning remain uncharacterized. To address this, we used fiber photometry with genetically encoded sensors [36, 37, 38, 39] to monitor stimulus‐evoked release of four neuromodulators in the OT. This enabled us to map their distinct dynamics across diverse contexts—including sex, internal states, and learning paradigms—yielding a comprehensive profile of their responses to rewards and associative cues.

## 2 Results

### 2.1 Distinct Release Patterns of OT Neuromodulators by Reward

**FIGURE 1:**
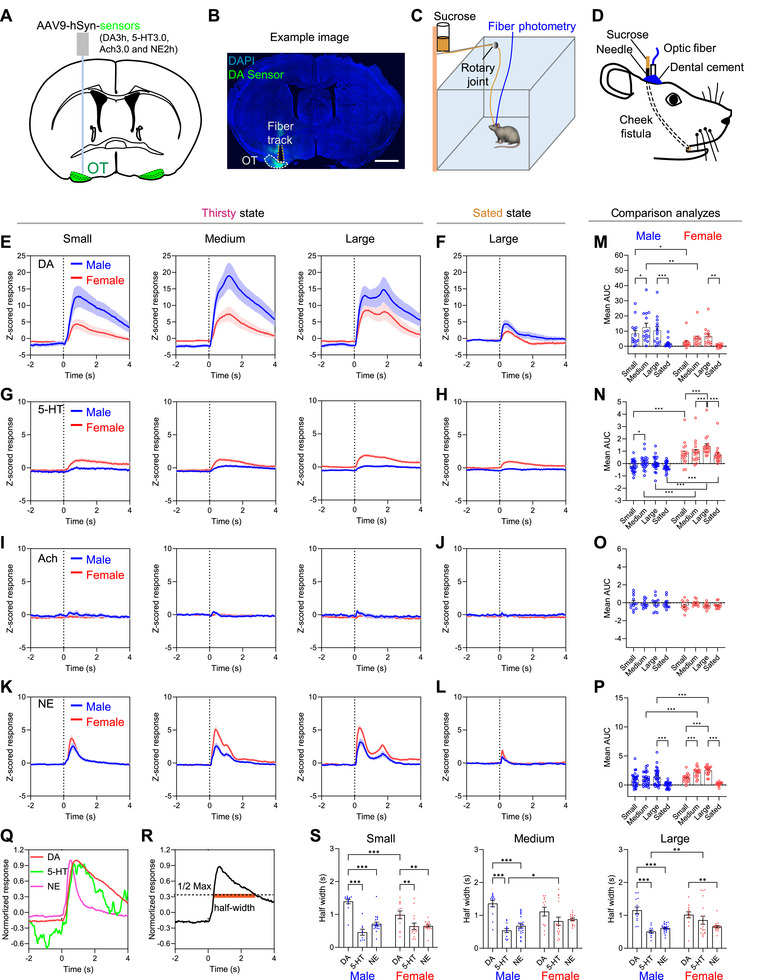
Sex‐specific, state‐dependent, and type‐selective responses of neuromodulators in the olfactory tubercle to external reward. (A) Schematic showing viral injection and fiber implantation for fiber photometry of genetically encoded neuromodulator sensors for DA (AAV9‐hSyn‐DA3h; D2R as the receptor backbone), 5‐HT (AAV9‐hSyn‐5‐HT3.0; 5HTR4 as the receptor backbone), Ach (AAV9‐hSyn‐Ach3.0; M3R as the receptor backbone), and NE (AAV9‐hSyn‐NE2h; α2AR as the receptor backbone) in the OT. Green areas delineate the olfactory tubercle (OT). (B) Example image of injection site and DA sensor expression in the OT of C57 animals. Orange dashed line shows fiber track; white dashed line delineates the OT. Scale bar, 1 mm. (C) Experiment diagram of reward delivery and fiber photometry recording in a freely‐moving mouse. (D) Schematic illustrating the intra‐oral solution infusion through a cheek fistula. (E–L) Peri‐stimulus time histograms (PSTHs) of average OT DA (E, F), 5‐HT (G, H), Ach (I, J), and NE (K, L) changes in males (blue) and females (red) mice. During thirst, with different reward magnitudes (E, G, I, and K). During satiety, with the large reward magnitude (F, H, J, and L). The time window is from 2 s before to 4 s after reward delivery. Thick lines indicate the mean, shaded areas indicate the SEM, and the light gray lines indicate the onset of sucrose reward. Black vertical dashed lines mark reward delivery onset (0 s). (M–P) Comparative analysis of the mean area under the curve (AUC) of DA (M), 5‐HT (N), Ach (O) and NE (P) under different conditions between males (blue) and females (red) within a 2‐s time window following reward onset. Two‐way repeated‐measures ANOVA with Bonferroni post‐hoc correction (^*^ *P* < 0.05, ^**^ *P* < 0.01, and ^***^ *P* < 0.001). Data is shown as mean ± SEM. (Q) Normalized reward response for OT DA (red), 5‐HT (green), and NE (purple). (R) Schematic of time window of 1/2 max response after reward onset (T1/2 max). Solid orange line represents the half‐width. Black dashed line stands for 1/2 max response. (S) Comparative analysis of half‐widths of reward responses for DA, 5‐HT, and NE across different reward magnitudes and sexes. Half‐widths were calculated only from those mice with significant responses. Two‐way ANOVA with Bonferroni post‐hoc correction (^*^ *P* < 0.05, ^**^ *P* < 0.01, and ^***^ *P* < 0.001). Data is shown as mean ± SEM. In (E, F, and M), n = 14 males and 13 females; in (G, H, and N), n = 23 males and 18 females; in (I, J, and O), n = 12 males and 12 females; and in (K, L, and P), n = 28 males and 17 females. For detailed statistical information, see Table S1 (Supporting Information).

To precisely monitor how OT neuromodulators (e.g., DA, 5‐H, ACh, and NE) encode external rewards in freely‐moving mice, we employed fiber photometry, an approach that is widely used to measure bulk neural dynamics in live animals with genetically encoded sensors [40]. These sensors were developed based on coupling the conformational changes of G protein‐coupled receptors (GPCRs) upon ligand (neurotransmitter/neuromodulator) binding with changes in the fluorescence signals of fluorescent proteins, thus using fluorescence imaging to detect neurotransmitter or neuromodulator release [1, 5, 36, 37, 38, 39]. We unilaterally injected AAV expressing these fluorescent neuromodulator indicators (DA [36]: AAV9‐hSyn‐DA3h and D2R as the receptor backbone; 5‐HT [38]: AAV9‐hSyn‐5‐HT3.0 and 5HTR4 as the receptor backbone; Ach [39]: AAV9‐hSyn‐Ach3.0 and M3R as the receptor backbone; and NE [37]: AAV9‐hSyn‐NE2h and α2AR as the receptor backbone) into the OT and implanted an optic fiber above the injection site (Figure 1A,B). Post hoc analysis suggested that genetically‐encoded sensors expression and optic fiber tips were largely restricted within the OT (Figure 1B and Figure S1). A sucrose reward was delivered randomly into the mouse's oral cavity via a cheek fistula (Figure 1C,D). Given that reward processing is influenced by factors like magnitude, internal states, and sex [18, 19, 20]. We designed experiments to explore their impact on OT neuromodulator dynamics. Our results revealed several key findings. First, reward presentation evoked distinct dynamics for each of the four neuromodulators: DA exhibited a biphasic response with a rapid rise and a slow decay (Figure 1E,F); 5‐HT showed a tonic activation (Figure 1G,H); ACh displayed no significant change but with a strong spontaneous release (Figure 1I,J; Figure S2); and NE responded with a phasic activation (Figure 1K,L). To examine that ACh responses were not significantly detected during reward is due to sensor specificity or a genuine biological phenomenon, we conducted an experiment using an aversive random foot‐shock paradigm. Our data showed that aversive stimuli (e.g., foot‐shock) triggered a robust, time‐locked Ach release (Figure S3), suggesting that Ach release is stimulus‐specific. In contrast to sensors, no significant change in fluorescence was observed in the GFP‐expressing control (Figure S4). Second, DA release was modulated by reward size, internal state, and sex (Figure 1E,F,M). Under thirst conditions, a medium reward evoked a greater DA response than a small reward in males. In a sated state, a large reward elicited less DA release in both sexes compared to the thirsty state. Notably, small and medium rewards provoked stronger DA release in males than in females. Third, 5‐HT release was also modulated by these factors (Figure 1G,H,N). Under thirst conditions, males showed a greater 5‐HT response to a medium reward than a small one, whereas females showed a greater response to a large reward. During satiety, a large reward evoked less 5‐HT release in both sexes compared to the thirst state. Overall, under identical conditions, reward‐evoked 5‐HT release was lower in males than in females. Fourth, reward delivery did not evoke significant Ach release under any condition (Figure 1I,J,O). Fourth, NE release was subject to a complex, sex‐selective modulation by both reward size and physiological state (Figure 1K,L,P). Under thirst conditions, medium and large rewards evoked greater NE release than small rewards in females, but this effect was absent in males. During satiety, a large reward elicited less NE release in both sexes compared to the thirst state. Notably, under thirst, males exhibited lower NE release than females in response to medium and large rewards. Finally, we analyzed the reward response half‐widths during thirst (Figure 1Q,R). DA release exhibited a significantly broader half‐width than NE in both sexes. DA responses were also broader than 5‐HT responses across all reward magnitudes in males, though in females this was true only for small rewards. Furthermore, females displayed significantly broader 5‐HT response half‐widths than males for medium and large rewards. Together, OT neuromodulator responses to reward are sex‐specific, state‐dependent, and subtype‐selective.

### 2.2 OT Neuromodulator Dynamics During Reward Learning, Extinction, and Re‐learning

**FIGURE 2:**
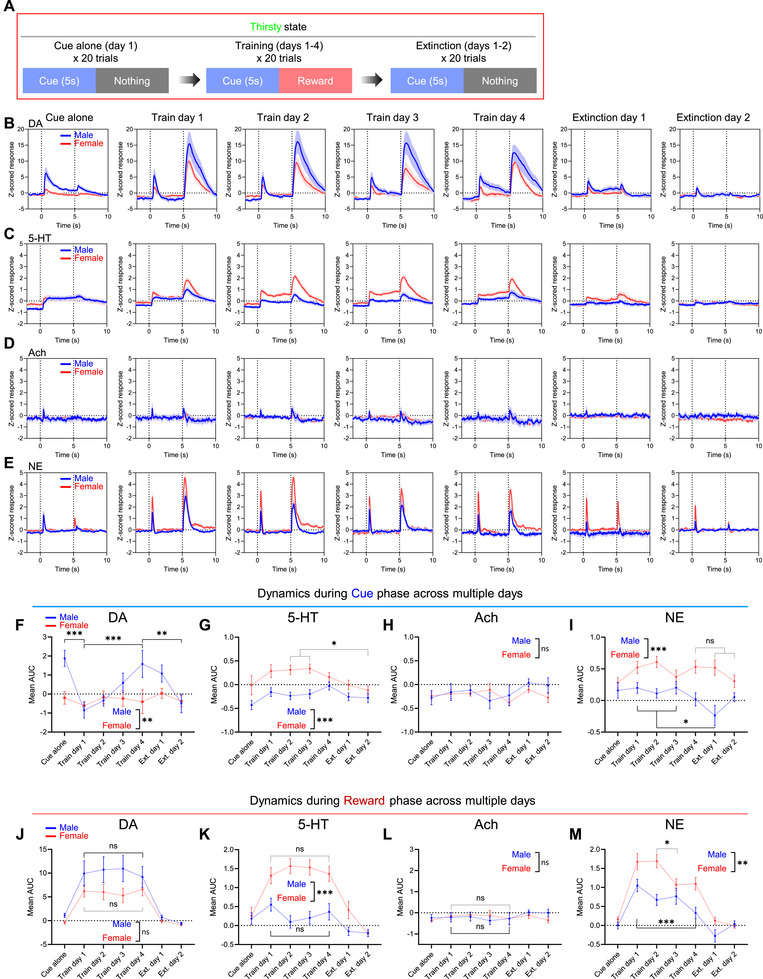
Sexually dimorphic encoding of reward learning and extinction by neuromodulators in the OT. (A) Experimental timeline focusing on reward learning and extinction: Mice underwent 20 trials/day across three phases: a 1‐day cue‐alone test (cue only), 4‐day training (cue + 0.5 s reward), and a 2‐day extinction phase (cue only). (B–E) PSTHs representing average changes in DA, 5‐HT, Ach, and NE activity across the three experimental phases in males (blue) and females (red). Each trial was initiated by a 5‐s auditory cue. Activity is shown from 2 s before to 10 s after cue onset (time = 0). Thick lines represent the mean, shaded areas represent the SEM, and black vertical dashed lines mark cue onset (0 s) and reward delivery (+5 s). (F–I) The mean AUC activity during the cue period. Mean AUC responses for DA, 5‐HT, Ach, and NE were calculated within a 0–5 s window for DA, 5‐HT and Ach and a 0–2 s window for NE following cue onset and compared across sexes (male: blue, female: red) and phases throughout the experiment. Data is shown as mean ± SEM. Statistical analysis was performed using a two‐way repeated‐measures ANOVA followed by Bonferroni's post‐hoc test (^*^ *P* < 0.05, ^**^ *P* < 0.01, and ^***^ *P* < 0.001; ns, not significant). Black lines label significant levels for males and gray lines for females. (J–M) The mean AUC activity during the period of reward delivery. Mean AUC responses for DA, 5‐HT, Ach, and NE were calculated within a 0–2 s window following reward onset and compared across sexes (male: blue, female: red) and phases throughout the experiment. Data is shown as mean ± SEM. Statistical analysis was performed using a two‐way repeated‐measures ANOVA followed by Bonferroni's post‐hoc test with a focus on comparisons across 4‐day training phases (^*^ *P* < 0.05, ^**^ *P* < 0.01, and ^***^ *P* < 0.001; ns, not significant). Black lines label significant levels for males and gray lines for females. In (B, F, and J), n = 14 males and 13 females; in (C, G, and K), n = 23 males and 18 females; in (D, H, and L), n = 11 males and 12 females; and in (E, I, and M), n = 28 males and 17 females. For detailed statistical information, see Table S1.

While neuromodulators are known to be powerful regulators of associative learning [1, 2, 3, 4], their synergistic or antagonistic interactions during the formation of new associations are poorly understood. Directly investigating these questions has been challenging because we lack the technology to track the dynamic release of multiple neuromodulators in vivo within a defined brain region. Here, we employed genetically encoded sensors for neuromodulators to examine how these OT neuromodulators dynamically track during reward learning and extinction (Figure 2A). To establish a clean baseline and rule out alternative explanations for our learning experiment results, we first performed a “cue alone” habituation experiment. We found that the cue (tone) alone elicited significant activation in DA, 5‐HT, and NE (Figure 2B–I). A trial‐by‐trial analysis revealed that these activation responses gradually decreased over successive trials, indicating habituation (Figure S4). Distinct neuromodulator dynamics were observed during the training phase cue period (Figure 2B–M). DA responses were phasic, evolving into a biphasic pattern in males but remaining stable in females, while 5‐HT showed a tonic activation that was more pronounced in females (Figure 2B,C,F,G). ACh displayed no significant change, and NE exhibited a phasic activation that grew in amplitude over training in females but not in males (Figure 2D,E,H,I). During the reward period, the absence of change in DA and 5‐HT suggests they do not encode reward prediction error in the OT, in contrast to NE, which showed a phasically decreasing response (Figure 2B,C,E,J,K,M). Finally, during extinction, cue‐evoked responses were markedly reduced or absent for all neuromodulators (Figure 2B–M).

**FIGURE 3:**
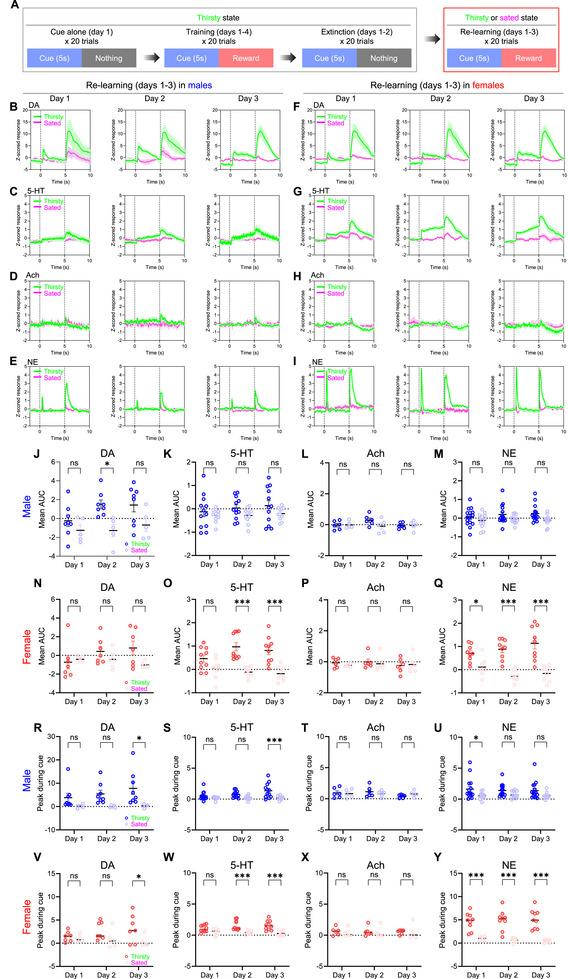
State‐dependent neuromodulator dynamics during reward re‐learning. (A) Experimental timeline focusing on re‐learning. Mice that have undergone reward learning and extinction were randomly divided into a thirsty group and a sated group. Mice underwent 20 trials/day as performed in reward learning. (B‐I) PSTHs representing average changes in DA (B, F), 5‐HT (C, G), Ach (D, H), and NE (E, I) activity during reward re‐learning in male (B‐E) and female (F‐I) mice. Green line stands for thirsty mice and the purple line for sated mice. Each trial was initiated by a 5‐s auditory cue. Activity is shown from 2 s before to 10 s after cue onset (time = 0). Thick lines represent the mean, shaded areas represent the SEM, and black vertical dashed lines mark cue onset (0 s) and reward delivery (+5 s). (J,Q) The mean AUC activity during the cue period of re‐learning. Mean AUC responses for DA (J, N), 5‐HT (K, O), and Ach (L, P) within a 0–5 s window and NE (M, Q) within a 0–2 s window following cue onset were calculated and compared across states (thirsty versus sated) throughout the experiment. In males, dark blue circles stand for the mean response in individual thirsty mice and light blue ones in sated mice; in females, dark red circles stand for the mean response in thirsty mice and light red ones in sated mice. Statistical analysis was performed using a two‐way repeated‐measures ANOVA followed by Bonferroni's post‐hoc test (^*^ *p* < 0.05, ^**^ *p* < 0.01, and ^***^ *p* < 0.001). Data is shown as mean ± SEM. The solid black line represents the mean. (R–Y) The mean AUC activity during the cue period of re‐learning. Peak responses for DA (R, V), 5‐HT (S, W), Ach (T, X), and NE (U, Y) were calculated within a 0–5 s window following cue onset and compared across states (thirsty versus sated) throughout the experiment. In males, dark blue circles stand for the mean response in individual thirsty mice and light blue ones in sated mice; in females, dark red circles stand for the mean response in thirsty mice and light red ones in sated mice. Statistical analysis was performed using a two‐way repeated‐measures ANOVA followed by Bonferroni's post‐hoc test (^*^ *p* < 0.05, ^**^ *p* < 0.01, and ^***^ *p* < 0.001). Data is shown as mean ± SEM. The solid black line represents the mean. In (B, J, R), n = 8 thirsty and 5 sated males; in (C, K, S), n = 12 thirsty and 11 sated males; in (D, L, T), n = 6 thirsty and 5 sated males; in (E, M, U), n = 15 thirsty and 13 sated males; in (F, N, V), n = 7 thirsty and 6 sated females; in (G, O, W), n = 10 thirsty and 8 sated females; in (H, P, X), n = 6 thirsty and 6 sated females; in (I, Q, Y), n = 9 thirsty and 8 sated females. For detailed statistical information, see Table S1.

Next, based on our finding that internal state profoundly modulates OT neuromodulator dynamics in response to reward (Figure 1), we next investigated its role during learning. To this end, we randomly assigned mice that had completed reward learning and extinction to one of two internal state groups—thirsty or sated—for a 3‐day reward re‐learning paradigm (Figure 3A). Remarkably, during the cue period of re‐learning (days 2–3), the sound cue evoked significantly greater responses in the thirsty group compared to the sated group for all neuromodulators except ACh; this was observed in both sexes (Figure 3B–Y), indicating that physiological state is a major regulator of learning. However, it also exhibits sex differences with a greater influence for DA release in males but for 5‐HT and NE release in females, suggesting a sex‐selective role in internal state modulation.

### 2.3 Dynamic Tracking of Cue Discrimination and Reversal Learning by OT Neuromodulators

**FIGURE 4:**
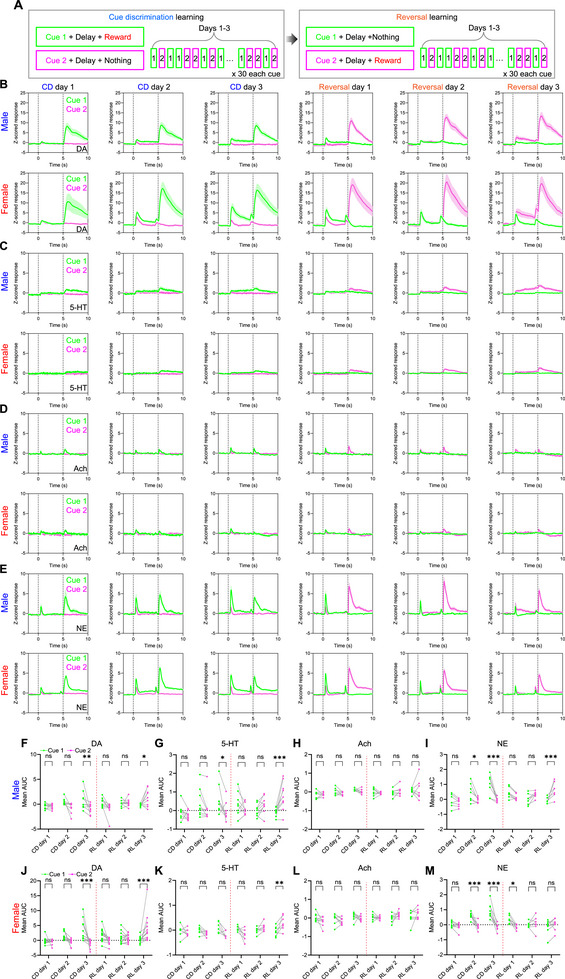
Dynamic and flexible tracking of cue discrimination and reversal learning by OT neuromodulators. (A) Time pipeline of experimental design. (B–E) PSTHs representing average changes in DA, 5‐HT, Ach, and NE activity across cue discrimination learning (CD days 1–3) and reversal learning (Reversal days 1–3) in males (upper) and females (lower). Each trial was initiated by a 4‐second auditory cue following 1‐s delay and 0.5 s reward delivery. Activity is shown from 2 s before to 10 s after cue onset (time = 0). Thick lines (green for Cue1 and purple for Cue 2) represent the mean, shaded areas represent the SEM, and black vertical dashed lines mark cue onset (0 s) and reward delivery (+5 s), respectively. (F–M) The mean AUC activity during cue period in males (F–I) and females (J–M). Mean AUC responses for DA (F, J), 5‐HT (G, K), Ach (H, L), and NE (I, M) were calculated within a 0–5 s window following cue onset and compared across cue discrimination and reversal phases. Green dots represent the mean response during the cue period of Cue 1, and purple ones for that of Cue 2. Red vertical dashed lines mark the onset of reversal. Statistical analysis was performed using a two‐way repeated‐measures ANOVA followed by Bonferroni's post‐hoc test (^*^ *p* < 0.05, ^**^ *p* < 0.01, and ^***^ *p* < 0.001). In (B, F, and J), n = 9 males and 13 females; in (C, G, and K), n = 9 male and 7 females; in (D, H, and L), n = 7 males and 8 females; and in (E, I, and M), n = 7 males and 8 females. For detailed statistical information, see Table S1.

Cognitive flexibility—the ability to adapt to changing environmental demands—is critical for survival and is typically assessed with reversal learning tasks [41, 42]. While OT neurons are known to flexibly encode reversals in stimulus valence [30], the contribution of OT neuromodulatory systems remains unknown. To investigate this, we conducted a behavioral session in which we reversed the sound‐outcome contingencies, making a previously rewarded sound unrewarded and a previously unrewarded sound rewarded (Figure 4A). We found that DA and 5‐HT release in the OT increased selectively during the rewarded sound cue in the discrimination phase, a pattern that reversed during the reversal phase as release decreased for the now‐unrewarded sound and increased for the newly rewarded one (Figure 4B,C,F,G,J,K). In contrast, NE release was sexually dimorphic (Figure 4E,I,M): although it increased during the rewarded cue in both sexes during discrimination, only males reversed this pattern during the reversal phase. ACh displayed no significant change (Figure 4D,H,L). Together, these findings demonstrate that OT neuromodulators dynamically track and encode changes in associative valence.

## 3 Discussion

While numerous studies have advanced our understanding of neuromodulator dynamics in associative learning and decision‐making, most have focused narrowly on one or two neuromodulators, primarily in males [6, 7, 8, 9, 10, 12, 15, 16, 17, 42, 43, 44, 45]. Given the potential for variation based on sex, reward properties, internal state, and behavioral context, a more comprehensive investigation is needed. Here, we combined fiber photometry with genetically encoded sensors to systematically capture reward‐evoked release patterns of four neuromodulators in the OT—an understudied component of the ventral striatum. We tracked their dynamics across distinct learning processes, including Pavlovian and reversal learning. Collectively, our findings provide a substantial and novel contribution to the field.

Our data uncovers sex‐specific, state‐dependent, and type‐selective response dynamics by systematically mapping the reward response profiles of four neuromodulators in the OT. Neuromodulators are key regulators of neuronal plasticity and behavior [1, 5]. Their actions are predominantly mediated by G protein–coupled receptors (GPCRs), which distinguishes their slow, pervasive signaling from the speed and precision of fast‐acting neurotransmitters [1, 5]. Indeed, many brain regions involved in motivation and learning—particularly the striatum—have been studied for their diverse neuromodulator dynamics and interactions [6, 7, 8, 9, 10, 11, 12, 13, 14, 15, 16, 43, 46]. Among the subregions of the striatum, the dorsal striatum and the NAc have received considerable attention [6, 7, 8, 9, 10, 11, 12, 13, 15, 16, 43, 46], whereas the OT remains largely unexplored, despite its known involvement in numerous behaviors [23, 24, 25, 26, 27, 28, 29, 30, 31, 32]. Our results demonstrate that reward evokes distinct dynamics for each of the four neuromodulators measured in the OT (Figure 1). These findings align with previous work on the functional diversity of neuromodulators [1, 5], highlighting their unique evolutionary roles, neural circuitry, and survival priorities. Collectively, this work and prior studies [6, 7, 8, 9, 10, 11, 12, 13, 15, 16, 43, 46] have begun to characterize the dynamics of neuromodulators underlying reward representation across various striatal subregions.

Specifically, DA exhibited a biphasic release pattern characterized by a rapid rise followed by a slow decay, consistent with previous studies of the reward response pattern of DA neurons in the VTA [40] and dopamine dynamics in the dorsal striatum and the NAc [8, 9, 11, 13, 15, 16]. This pattern reflects a dual‐timescale mechanism, optimized for initial reward detection and sustained motivational persistence. Critically, emerging evidence indicates that a blunted phasic DA response (a reduced initial peak) impairs reward detection, while a depletion in tonic DA (a flattened slow phase) is associated with anhedonia [17, 47, 48]. Given the OT's established involvement in addiction and depression [29, 31], this collective evidence suggests that the OT's specific DA response pattern to reward may serve as a potential biological marker for these and other neuropsychiatric disorders characterized by reward dysfunction. In addition, we found that 5‐HT in the OT showed a sustained, tonic activation by reward, likely acting as a long‐term emotional and motivational regulator [4, 49, 50]. Interestingly, our observation is consistent with previous studies of the reward response pattern of 5‐HT neurons in the dorsal raphe nucleus (DRN) [22, 51]. Indeed, the OT receives dense serotonergic innervation from the ascending DRN serotonergic pathway. This slow, sustained 5‐HT modulation within the DRN‐OT circuit likely complements DA's fast signals, ensuring balanced reward‐driven behavior [49]. Furthermore, our data shows that NE responded with a phasic activation, likely acting as a neural alarm bell that enhances attention toward rewarding stimuli [2]. Although ACh release in the dorsal striatum and NAc is responsive to reward [13, 15, 52], it was not significantly evoked by reward in any condition in the OT, unlike the release of other neuromodulators. This lack of Ach response could be attributed to the specificity of our sensors, which target a single receptor type and may be inefficient for ACh, or it may reflect a true biological phenomenon where OT ACh release is specifically tuned to aversive rather than rewarding stimuli [53]. Indeed, aversive stimuli (e.g., foot‐shock) triggered a robust, time‐locked Ach release (Figure S3), suggesting that Ach release is stimulus‐specific and reflects a true biological phenomenon. Broadly, investigating how OT neuromodulators respond to both rewarding and aversive stimuli offers a compelling avenue for future research.

Notably, our results also reveal a sex‐specific and state‐dependent role for neuromodulators in reward processing. We observed stronger DA reward responses in males, contrasted by stronger 5‐HT and NE responses in females. These observed sex differences in reward processing may be attributable to several mechanisms. These include potential sex differences in the neuromodulatory projections to OT circuits, as well as differential expression of neuromodulator receptors within these pathways. Thus, fully understanding the underlying sex‐modulated mechanisms represents an interesting future research direction. Moreover, the activity of all three systems was also modulated by internal state and, to some extent, by reward magnitude. Prior research has established that neural dynamics can be controlled via dedicated hardwired circuits, which scale excitatory drive onto specific neurons according to need magnitude. Given the profound influence of neuromodulation on neural activity, our results complement this view by showing that neuromodulator dynamics are also subject to regulation by an individual's internal state. This architecture supports the precise and adaptive control of behavior necessary for physiological homeostasis. Overall, these findings are particularly significant as reward processing and its associated pathologies are known to exhibit profound sex differences and individual variation [1, 11, 18, 19, 20, 21, 22]. Consequently, our work provides novel insights into understanding of the functional roles of neuromodulators in both healthy and diseased states.

Moreover, our results demonstrate that OT neuromodulators encode reward learning, extinction, and re‐learning in a sexually dimorphic and state‐dependent manner. During the cue period of the learning phase, we observed distinct dynamic responses. We observed a phasic DA response to the cue that evolved into a biphasic pattern (rapid rise, slow decay). The phasic component is facilitated by low‐affinity receptors and rapid vesicle fusion, whereas the tonic component is proposed to extend the time window for synaptic plasticity to reinforce learning [47, 54]. This mechanism illustrates how DA effectively promotes both immediate learning and sustained motivation. Crucially, however, OT DA levels to reward did not significantly change across the four‐day training in either sex, diverging from the well‐established role of DA in encoding reward prediction error. Interestingly, this data is in line with dorsal striatal DA release to reward. Similar to the response of DRN 5‐HT neurons during reward learning [22, 47, 51, 54], 5‐HT release demonstrated sustained tonic activation that transitioned to a ramping pattern. This modulation possibly balances the fast phasic signals of DA to ensure stable reward‐driven learning [49]. Unlike the tonic activation of DA and 5‐HT, NE showed a phasic cue response that gradually grew stronger with training. Consistent with previous work on the locus coeruleus NE‐expressing neuronal activity during learned behavior [55], phasic NE involves the maintenance of alertness and arousal, thereby facilitating reward‐related learning [2]. During extinction, all neuromodulators showed markedly reduced or absent cue responses compared to the learning phase, indicating that their role in driving learning behavior is highly reward‐dependent. Furthermore, during reward re‐learning, internal states like thirst also profoundly reshaped OT neuromodulator dynamics and exhibits sex differences with a greater influence on DA release in males but on 5‐HT and NE release in females. These findings highlight the context‐dependent, sex‐specific, and dynamic role of neuromodulators in learning. Collectively, our results underscore the distinct yet synergistic roles that OT neuromodulators play in learning, demonstrating their potential to regulate neuronal activity underlying adaptive physiology and behavior.

Finally, we find that OT neuromodulators (DA, 5‐HT, and NE) dynamically encode reward contingency reversal through distinct activation patterns: tonic responses in DA and 5‐HT systems versus phasic responses in NE (Figure 4). This implicates them directly in behavioral flexibility, a critical cognitive process for survival known to involve switching neural activity across a distributed network [41]. Notably, NE responses reversed in males but not in females during reversal learning, a finding that may reflect sex‐differentiated cognitive strategies. Several possibilities could account for this sex difference. One possibility is that males and females engage distinct cognitive processes to solve the same task. Males may rely more on flexible, rule‐based strategies that require updating internal models when contingencies change—a process heavily dependent on norepinephrine (NE) signaling from the locus coeruleus (LC). In contrast, females may depend more on habitual or stimulus‐response strategies. If females do not recruit the same cognitive flexibility circuits, the phasic NE signal that tracks rule reversal may not be engaged. Alternatively, the absence of a reversal‐related NE signal in females may not reflect a lack of cognitive flexibility, but rather the involvement of a distinct neural circuit to execute the same behavior. Without simultaneous recording of multiple neuromodulators, the female brain may appear “NE silent” during reversal learning when, in fact, another system has assumed the lead role. Consistent with this idea, our findings build upon prior work showing that OT neurons themselves encode reversals [30]. We now expand this view by demonstrating that local neuromodulatory systems are also integral to this process. This suggests a dual mechanism in the OT, where fast neuronal activity is complemented by slower neuromodulatory signaling to govern adaptive behavior. This synergy solidifies the fundamental role of the OT in cognitive flexibility, illustrating how neuromodulators sculpt neuronal activity to integrate internal states like motivation and arousal with sensory input to guide learning and behavior.

A key methodological limitation is our use of sensors targeting single receptor type for each neuromodulator. Consequently, our data capture the activity of specific receptor pathways rather than total neuromodulator release. Furthermore, although cross‐animal comparisons revealed consistent response patterns to stimuli, our design did not permit the simultaneous measurement of multiple neuromodulators in a single subject, which has several significant impacts on our understanding of brain function. Firstly, it precludes the ability to assess real‐time interactions and synergistic dynamics among neuromodulators. Without simultaneous measurements, researchers cannot observe how different neuromodulators—such as dopamine and serotonin—directly influence each other's release during behavior. Given that neuromodulators do not function in isolation, this limitation obscures whether their relationships are synergistic, antagonistic, or permissive in specific behavioral contexts. Secondly, it prevents the detection of state‐dependent coding. The neuromodulatory state of the brain fundamentally shapes how neurons respond to incoming signals. Recording only one modulator omits this contextual information. For example, the same phasic dopamine release may signal “reward” in a high‐acetylcholine state but convey “salience” in a high‐norepinephrine state. Without a multi‐modulator perspective, it becomes impossible to understand how global neuromodulatory context gates information processing. Third, the lack of concurrent data hampers the development of computational models that incorporate multiple neuromodulators during behavior. Computational models depend on accurate parameters to simulate brain function. In the absence of simultaneous recordings, models cannot capture the covariance structure between neuromodulatory signals. This leads to oversimplified representations that treat modulatory inputs as independent variables rather than as coupled, interacting systems, ultimately limiting predictive power regarding behavior. Finally, this limitation increases biological variance and the required sample size. Comparing neuromodulators across different experimental conditions often necessitates separate groups of animals or sequential trials using different probes. This introduces inter‐individual variability—such as differences in stress, metabolism, or genetics—as a confounding factor. Consequently, larger sample sizes are needed to achieve statistical power, raising ethical concerns about the number of animals used. Given these challenges, achieving simultaneous recording of multiple neuromodulators within the same animal is of critical importance. To address this goal, color‐coded neuromodulator sensors have been developed and validated in various brain regions. Applying these next‐generation tools to monitor the dynamic patterns of distinct neuromodulators concurrently represents a promising and important direction for future research.

Another limitation arises from our reward delivery method—intra‐oral infusion via a cheek fistula. While ideal for precise reward control in Pavlovian paradigms, this approach decouples the natural link between action and outcome, preventing us from correlating neuromodulator dynamics with trial‐by‐trial behavioral performance. In fact, recent studies have begun to explore voluntary behavioral engagement (e.g., operant conditioning). For example, a recent study examined how dopamine and acetylcholine release during effortful behavior by using a sucrose forage task [56]. Therefore, a critical future direction is the simultaneous measurement of multiple neuromodulators in the OT during instrumental behavior within the same animal.

In addition, previous studies demonstrated that the medial and lateral part of the OT usually have completely opposite functions [26]. However, based on the viral injection sites for each animal (Figure S1B) in our study and the corresponding neuromodulator dynamics in response to reward (Figure 1), we did not observe an obvious correlation between modulatory activity patterns and anatomical location within the olfactory tubercle (OT). To my knowledge, this absence of observed correlation does not preclude the possibility of a direct link between neuromodulatory dynamics and regional structure. Several factors may account for this. First, given the differential expression of neuromodulator receptors across medial versus lateral OT and the functional diversity among receptor subtypes for a given neuromodulator [24, 26, 34], our use of sensors targeting only a single receptor type per neuromodulator limits our ability to capture this spatial and functional heterogeneity. Second, these neuromodulators do not operate in isolation but engage in complex cross‐talk through multiple presynaptic and postsynaptic mechanisms [1, 26]. By recording only one modulator at a time, our approach necessarily omits this regulatory dimension. Third, afferent projections from neuromodulator‐producing brain regions differentially target subregions of the OT, which may contribute to functional segregation—such as attraction versus aversion, or flexible versus rigid responding—between medial and lateral compartments. To directly test this framework, future studies employing subregion‐specific fiber photometry in combination with receptor‐selective pharmacological manipulations will be essential. Such approaches could reveal how the OT achieves its opposing functions through differential neuromodulatory encoding across its medial and lateral subdivisions.

In conclusion, our study delineates the dynamics of four reward‐evoked OT neuromodulators, capturing their roles in both reward processing and associated learning while accounting for sex and internal states. Future work should simultaneously monitor the interactions among these neuromodulators under diverse conditions to pinpoint the precise neural mechanisms of learning. Ultimately, decoding this complex interplay is crucial for developing treatments for related neurobiological disorders.

## 4 Experimental Section

Additional details for all methods are provided in the Methods.

To precisely monitor how OT neuromodulators (e.g., DA, 5‐H, ACh and NE) encode external rewards in freely‐moving mice considering many factors such as reward magnitude, sex, and internal, we employed fiber photometry, an approach that is widely used to measure bulk neural dynamics in live animals with genetically encoded sensors. Then, we examined how these OT neuromodulators dynamically track during reward learning, extinction, and re‐learning. Finally, we tested how these OT neuromodulators dynamically track during cue discrimination and reversal learning.

## Author Contributions

Conceptualization, R.K.H.; formal analysis, R.K.H. and M.H.; investigation, M.H., L.Z., Y.C., S.H., J.G.; technical support, Z.X. and T.M.; writing – review & editing, R.K.H. and S.H.; supervision, R.K.H.; funding acquisition, R.K.H and J.G.

## Conflicts of Interest

The authors declare no conflict of interest.

## Supporting information




**Supporting File 1**: advs74973‐sup‐0001‐SuppMat.docx.

**Supporting File 2**: advs74973‐sup‐0002‐Figures.pdf.

**Supporting File 3**: advs74973‐sup‐0003‐Tables.pdf.

## Data Availability

The data that support the findings of this study are available on request from the corresponding author. The data are not publicly available due to privacy or ethical restrictions.
