# Supplementary material for: Neuromodulator Dynamics Underlying Associative Learning in the Ventral Striatum's Olfactory Tubercle
Source: Adv Sci (Weinh). 2026 Mar 23;13(32):e74973. doi: 10.1002/advs.74973 (PMC13252644; doi:10.1002/advs.74973)
Supplement: Supplementary file 1 — Supporting File 1: advs74973‐sup‐0001‐SuppMat.docx. [file ADVS-13-e74973-s003.docx]

**Methods**

**Mice**

C57BL/6J mice (8-week-old, both males and females; GemPharmatech, China) were used for stereotaxic surgery and behavioral experiments. Animals were grouped housed in 12 h light-dark cycle (9 p.m. - 9 a.m. light), with food and water available ad libitum. Animal care and use were strictly followed according to the institutional guidelines and governmental regulations of China. Experiments were performed exactly as approved by the IACUC at Fudan University.

**Stereotaxic Surgeries**

For monitoring neuromodulator dynamics, 150 nl of genetically encoded sensors for dopamine (AAV9-hSyn-DA3h), serotonin (AAV9-hSyn-5-HT3.0), acetylcholine (AAV9-hSyn-Ach3.0), or norepinephrine (AAV9-hSyn-NE2h) were injected into the olfactory tubercle (OT) (AP: +0.25 mm, ML: ± 2.00 mm, DV: -5.90 mm from Bregma). An optical fiber was then implanted ~0.10 mm above the injection site. Same volume of AAV9-EF1ɑ-EYFP virus was injected into the OT as the controls. A sucrose reward was delivered into the mouse's oral cavity via a cheek fistula throughout all experiments in this study.

**Random reward**

We investigated how OT neuromodulator dynamics are influenced by reward magnitude, sex, and internal state using a 3% sucrose solution reward. All mice were water-restricted for 8-10 hours prior to experimentation. To evaluate reward magnitude, we administered small (0.2 s), medium (0.8 s), and large (1.5 s) rewards from thirsty mice across three consecutive days (Days 1-3). Reward delivery was controlled by a custom MATLAB program and an Arduino board, maintaining a constant flow rate of 100 µl/s. Each day, both male and female mice underwent 20 trials with a 40-50 second trial interval. Finally, on Day 4, we examined the effect of internal state by recording dynamics from sated mice exposed to the large reward. We calculated using Matlab ‘findpeaks’ function and compared half-widths of reward responses for DA, 5-HT and NE across different reward magnitudes and sexes. Half-widths were calculated only from those mice with significant responses. Because ACh responses were not significantly detected under almost all conditions. To confirm that this is a genuine biological phenomenon, we conducted an experiment using an aversive random foot-shock paradigm. Over three consecutive days (Days 1–3), mice received 1-second foot-shocks at three different intensities: small (0.2 mA), medium (0.6 mA), and large (1.0 mA). Foot-shock delivery was controlled by a custom MATLAB program interfaced with an Arduino board. Each daily session consisted of 6 trials, with a variable inter-trial interval of 40–50 seconds.

**Pavlovian** **reward learning, extinction and re-learning**

The procedure for Pavlovian reward learning and extinction is as follows: 1) Habituation (Days 1-3): Mice were placed in the open arena for free exploration to acclimate to the environment; 2) Cue alone (Day 4), a sound cue at the frequency of 7000 Hz was presented for 5 s and after which no sucrose reward was given during a 5 s reward period. Mice underwent 20 trials, with a trial-interval of 40-50 seconds; 3) Learning (Days 5-8): a sound cue at the frequency of 7000 Hz was presented for 5 s, followed by a 0.5 s continuous reward delivery. Mice underwent 20 trials, with a trial-interval of 40-50 seconds; 4) Extinction (Days 9-10): similar with the parameters used in Cue alone. OT neuromodulator dynamics was monitored across the phases of Cue alone, Conditioning and Extinction. We next investigated the role of internal state in neuromodulator during re-learning. We randomly assigned mice that had completed reward learning and extinction to one of two internal state groups—thirsty or sated—for a 3-day reward re-learning paradigm, similar with that previously used in reward learning.

**Cue discrimination and reversal learning**

To investigate the contribution of OT neuromodulatory systems during reversals, we conducted a behavioral session in which we reversed the sound-outcome contingencies, making a previously rewarded sound unrewarded and a previously unrewarded sound rewarded. During cue discrimination (Days 1-3), Cue 1 (7000 Hz tone) is paired with 0.5 s reward with a 1-s delay between the cue and reward and Cue 2 (2000 Hz tone) with 0.5 s time-out. Each daily training session typically consists of 30 trials for Cue 1 and 30 trials for Cue 2, with a trial interval of 40-50 seconds. Cue 1 and Cue 2 were presented in a randomized manner with no more than three consecutive repetitions of the same trial type. During reversal learning (Days 4-6), only the cue and reward conditions were reversed: mice learn that the previously rewarded cue no longer yields a reward, while the previously unrewarded cue now predicts an upcoming reward.

**Fiber photometry experiments and data analysis**

Viral microinjections of AAVs encoding neuromodulator sensors were targeted to the olfactory tubercle (OT). Fluorescence signals were acquired using multi-channel fiber photometry systems (Inper, China) with 410/470 nm or 470/561 nm wavelength configurations. To record sensor activity, light from 470 nm LEDs was bandpass-filtered (470/10 nm) for excitation. For the 410/470 system, a 410 nm LED served as an isosbestic control and was delivered alternately with the 470 nm excitation light. Similarly, for the 470/561 system, a 561 nm LED was used as the isosbestic control channel and alternated with the 470 nm light. The LED power was adjusted to 30–50 µW at the optical fiber tip to minimize photobleaching. Signals were imaged from the fiber tip at a sampling rate of 100 fps and acquired using the Inper Signal software (Inper, China). Prior to behavioral testing, mice were habituated to the attached optic fiber cable. Fluorescence signals were processed as follows. The normalized fluorescence change was calculated as: Fn(t) = 100 × (F_470_(t) – F_405_fit(t)) / mean(F_405_fit(baseline_period)) for the 410/470 system, or Fn(t) = 100 × (F_470_(t) − F_561_fit(t)) / mean(F_561_fit(baseline_period)) for the 470/561 system, where F_405_fit(t) and F_561_fit(t) represent the fitted isosbestic control signals and baseline_period refers to a 1 s window between 5 s and 4 s before each event/stimulus onset. For population analysis, traces were z-score normalized before averaging. Heatmaps and average sensor traces were generated using a custom MATLAB script, with shaded areas on trace plots indicating the standard error of the mean (s.e.m.).

**Histology and Imaging**

After the experiment, mice were perfused with 1× phosphate-buffered saline (1× PBS) for approximately 5 minutes, followed by perfusion with 4% paraformaldehyde (PFA) for 10–15 minutes. Brain tissues were post-fixed with 4% PFA at room temperature for 2 hours, and then transferred to a 30% sucrose solution for dehydration overnight at 4°C. To visualize viral expression and optic fiber location, brain tissues were sectioned into 40-micrometer (µm) thick using a cryostat (RWD Life Science). To strengthen the fluorescent signals in the OT, GFP immunofluorescence staining was performed on the brain slices containing the OT. The 40 µm brain slices were incubated with rabbit anti-GFP primary antibody (Abcam, Cat. No. ab290, dilution 1:1000) overnight at 4°C. The same brain slices were incubated with goat anti-rabbit Alexa 488 fluorescent secondary antibody (Abcam, ab150077, dilution 1:1000) at room temperature for 2 hours. Brain slices of interest were imaged using a confocal microscope (Olympus, Model FV3000) to obtain high-resolution fluorescence images for subsequent analysis and verification.

**Data analysis and statistics**

No statistical methods were applied to pre-determine sample size. Data collection and analysis was performed blind to the conditions of experiments. Figures were plotted using Prism version 10 (GraphPad) or MATLAB 2021a (MathWorks). Data is reported as dot-line plots, or mean ± SEM plots. Statistical methods used in this study include two-way repeated measures ANOVA. The data met the assumptions of the statistical tests used. Normality of the data was tested using the Kolmogorov-Smirnov test. Statistically significant differences were established at *P < 0.05, **P < 0.01 and ***P < 0.001; n.s. indicates not significant.
