# Supplementary material for: Neuromodulator Dynamics Underlying Associative Learning in the Ventral Striatum's Olfactory Tubercle
Source: Adv Sci (Weinh). 2026 Mar 23;13(32):e74973. doi: 10.1002/advs.74973 (PMC13252644; doi:10.1002/advs.74973)
Supplement: Supplementary file 2 — Supporting File 2: advs74973‐sup‐0002‐Figures.pdf. [file ADVS-13-e74973-s001.pdf]

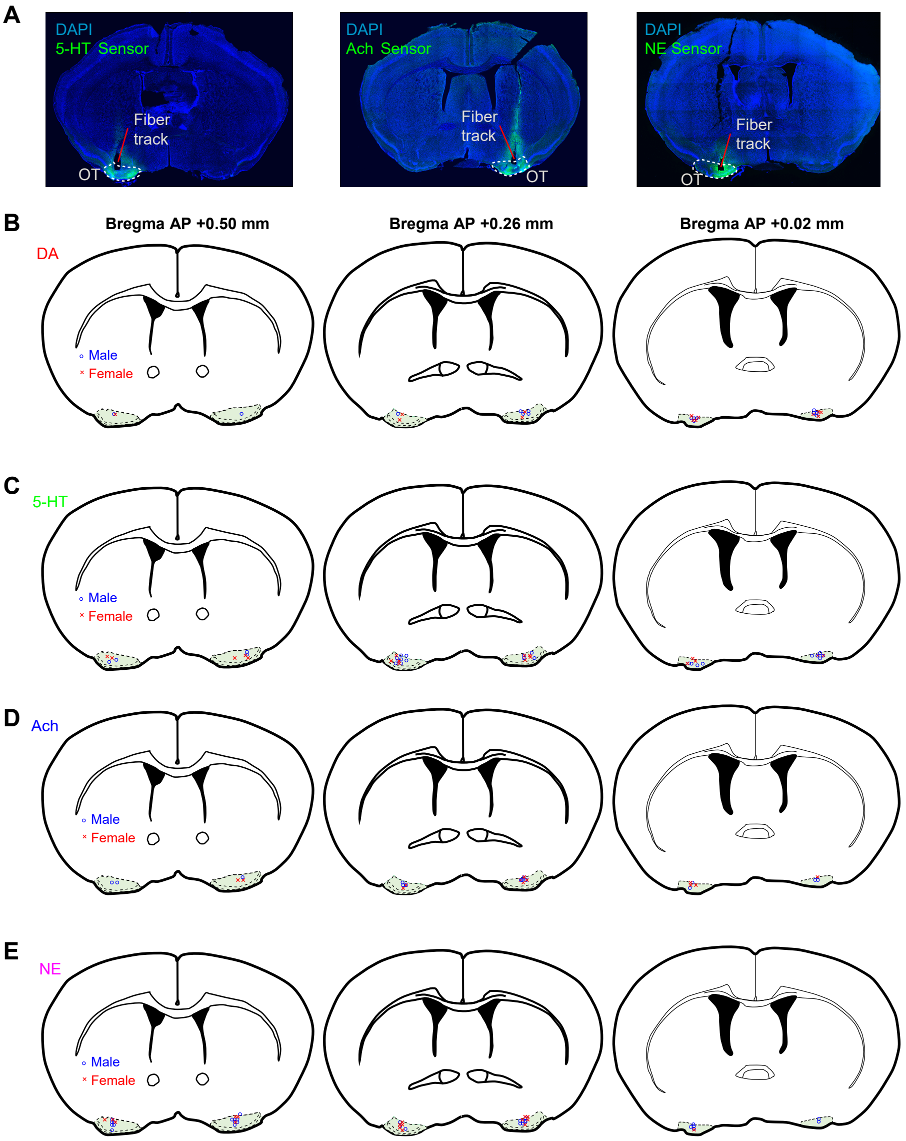

Figure S1. (A) Example images of injection sites and expression profiles for 5-HT, Ach and NE in the OT of C57 animals. White dashed line delineates the OT. Scale bar, 1 mm. (B-E) Optic fiber tip locations for DA (B), 5-HT (C), Ach (D) and NE (E) male (blue) and female (red) mice.

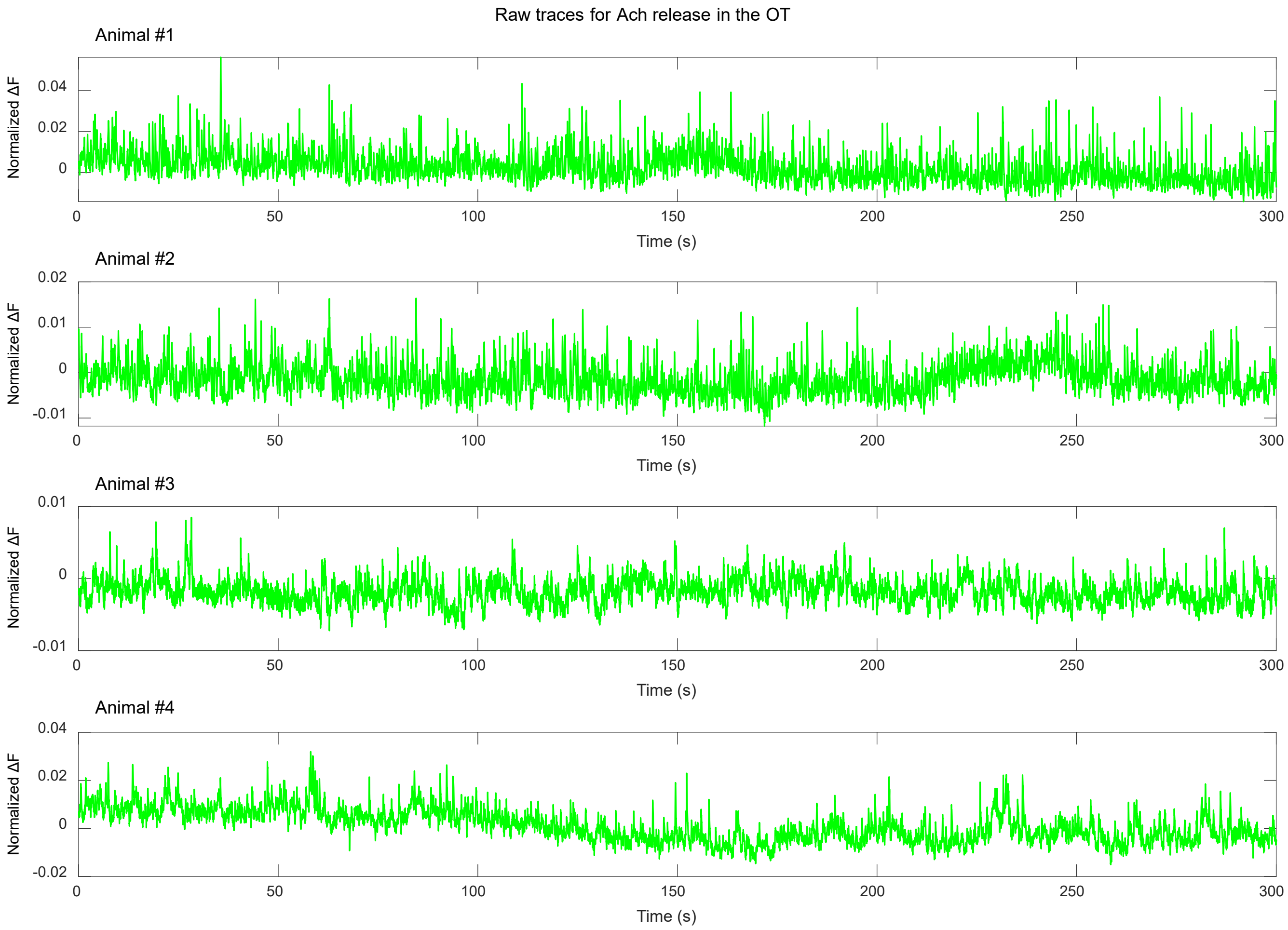

Figure S2. Example raw traces of spontaneous Ach release in the OT.

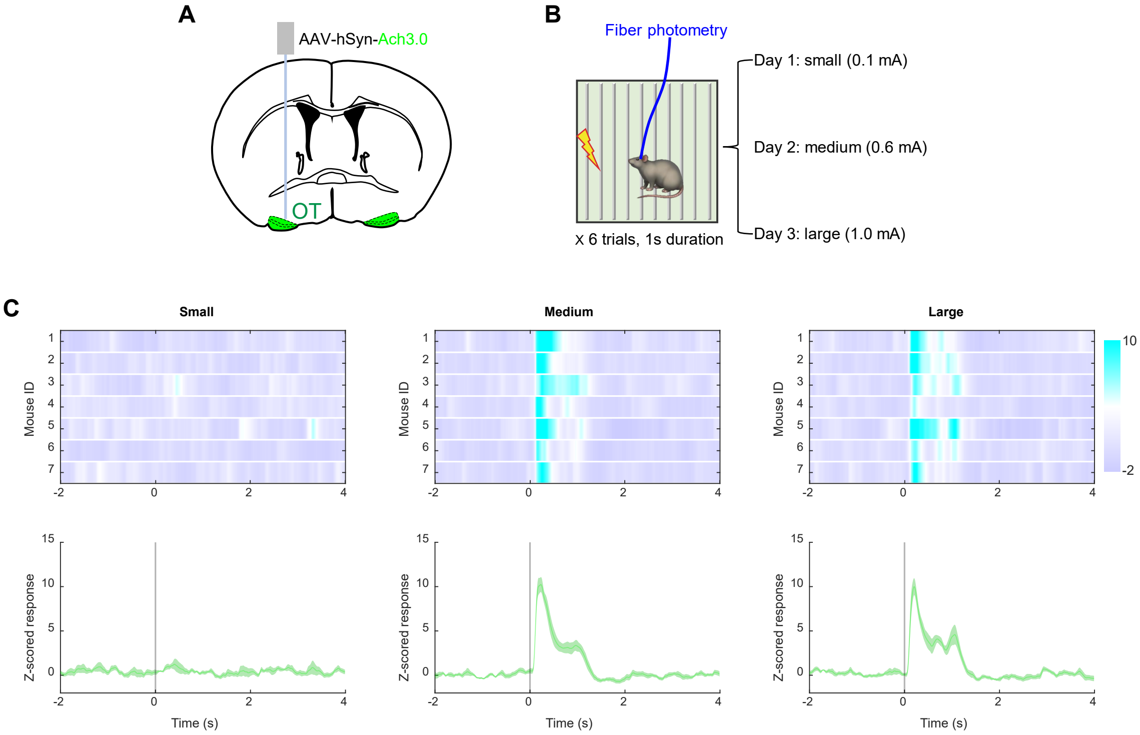

Figure S3. Foot-shocks induced robust time-locked Ach release in the OT.

(A) Schematic showing viral injection and fiber implantation for fiber photometry of genetically encoded neuromodulator sensors Ach (AAV9-hSyn-Ach3.0; M3R as the receptor backbone) in the OT. Green areas delineate the olfactory tubercle (OT).

(B) Experiment diagram of foot-shock delivery and fiber photometry recording in a freely-moving mouse. It consisted of a three-day consecutive test for recording Ach release at three different intensities (small, 0.1 mA; medium, 0.6 mA; large, 1.0 mA).

(C) Heatmaps (upper) of mean responses for each animal and PSTHs (lower) of average Ach release by aversive foot-shocks at three different intensities over three consecutive days (Days 1–3). Foot-shock triggered a robust, time-locked Ach release in the OT.

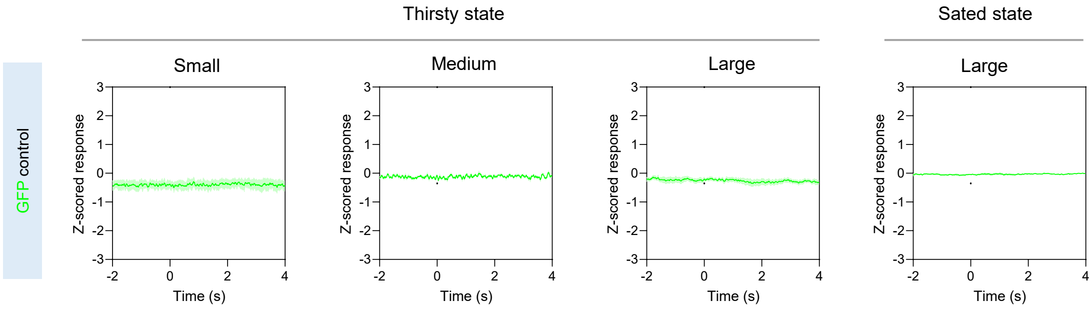

Figure S4. Average PSTHs of GFP-expressing control show no detectable fluorescent changes across conditions.
