# Supplementary material for: Neuromodulator Dynamics Underlying Associative Learning in the Ventral Striatum's Olfactory Tubercle
Source: Adv Sci (Weinh). 2026 Mar 23;13(32):e74973. doi: 10.1002/advs.74973 (PMC13252644; doi:10.1002/advs.74973)
Supplement: Supplementary file 3 — Supporting File 3: advs74973‐sup‐0003‐Tables.pdf. [file ADVS-13-e74973-s002.pdf]

Fig	Sample Size	Analysis (all tests are two-sided)	Statistical Test Values
1M	DA:<br>N = 14 male;<br>N = 13 female	Two-way repeated-measures ANOVA with Bonferroni post-hoc correction.<br>Column factor: sexes (male vs female)<br>Row factor: reward sizes	Reward sizes: F (3, 75) = 20.7, ***p<0.001<br>Sexes: F (1, 25) = 4.28, *p=0.049<br>Bonferroni's multiple comparison test:<br>1. Male vs. Female:<br>Small: *p=0.030<br>Medium: ** P=0.006<br>Large: p=0.159<br>Sated: p=0.535<br><br>2. Among reward sizes:<br>Small vs. Medium: male, *p=0.049; female, p=0.711<br>Small vs. Large: male, p>0.999; female, p=0.079<br>Medium vs. Large: male, p=0.954; female, p>0.999<br>Large vs. Sated: male, ***p<0.001; female, **p=0.001
1N	5-HT:<br>N = 23 male;<br>N = 18 female	Two-way repeated-measures ANOVA with Bonferroni post-hoc correction.<br>Column factor: sexes (male vs female)<br>Row factor: reward sizes	Reward sizes: F (3, 117) = 15.8, ***p<0.001<br>Sexes: F (1, 39) = 31.7, ***p<0.001<br>Bonferroni's multiple comparison test:<br>1. Male vs. Female:<br>Small: ***p<0.001<br>Medium: ***p<0.001<br>Large: ***p<0.001<br>Sated: ***p<0.001<br><br>2. Among reward sizes:<br>Small vs. Medium: male, *p=0.015; female, p>0.999<br>Small vs. Large: male, p=0.216; female, ***p<0.001<br>Medium vs. Large: male, p>0.999; female, ***p<0.001<br>Large vs. Sated: male, p=0.124; female, ***p<0.001
1O	Ach:<br>N = 12 male;<br>N = 12 female	Two-way repeated-measures ANOVA with Bonferroni post-hoc correction.<br>Column factor: sexes (male vs female)<br>Row factor: reward sizes	Reward sizes: F (3, 66) = 0.520, p=0.670<br>Sexes: F (1, 22) = 1.49, p=0.235
1P	NE:<br>N = 28 male;<br>N = 17 female	Two-way repeated-measures ANOVA with Bonferroni post-hoc correction.<br>Column factor: sexes (male vs female)<br>Row factor: reward sizes	Reward sizes: F (3, 129) = 50.8, ***p<0.001<br>Sexes: F (1, 43) = 9.58, **p=0.003<br>Bonferroni's multiple comparison test:<br>1. Male vs. Female:<br>Small: p=0.339<br>Medium: ***p<0.001<br>Large: ***p<0.001<br>Sated: p=0.521<br><br>2. Among reward sizes:<br>Small vs. Medium: male, p>0.999; female, ***p<0.001<br>Small vs. Large: male, p=0.091; female, ***p<0.001<br>Medium vs. Large: male, p=0.529; female, p>0.999<br>Large vs. Sated: male, ***p<0.001; female, ***p<0.001
1S-left	Same animals used in 1N-P	Two-way ANOVA with Bonferroni post-hoc correction.<br>Column factor: sexes (male vs female)<br>Row factor: neuromodulator types	Interaction: F (2, 82) = 6.48, **p=0.002<br>Neuromodulator types: F (2, 82) = 35.7, ***p<0.001<br>Sexes: F (1, 82) = 2.35, p=0.129<br>Bonferroni's multiple comparison test:<br>1. Male vs. Female:<br>DA: ***p<0.001<br>5-HT: p=0.131<br>NE: p=0.475<br><br>2. Among neuromodulator types:<br>DA vs. 5-HT: male, ***p<0.001; female, **p=0.007<br>DA vs. NE: male, ***p<0.001; female, **p=0.006<br>5-HT vs. NE: male, p=0.078; female, p>0.999
1S-middle	Same animals used in 1N-P	Two-way ANOVA with Bonferroni post-hoc correction.<br>Column factor: sexes (male vs female)<br>Row factor: neuromodulator types	Interaction: F (2, 90) = 4.36, *p=0.016<br>Neuromodulator types: F (2, 90) = 18.9, ***p<0.001<br>Sexes: F (1, 90) = 1.16, p=0.285<br>Bonferroni's multiple comparison test:<br>1. Male vs. Female:<br>DA: p=0.087<br>5-HT: *p=0.033<br>NE: p=0.077<br><br>2. Among neuromodulator types:<br>DA vs. 5-HT: male, ***p<0.001; female, p=0.090<br>DA vs. NE: male, ***p<0.001; female, p=0.244<br>5-HT vs. NE: male, p=0.793; female, p>0.999
1S-right	Same animals used in 1N-P	Two-way ANOVA with Bonferroni post-hoc correction.<br>Column factor: sexes (male vs female)<br>Row factor: neuromodulator types	Interaction: F (2, 92) = 4.48, *p=0.014<br>Neuromodulator types: F (2, 92) = 19.2, ***p<0.001<br>Sexes: F (1, 92) = 1.78, p=0.185<br>Bonferroni's multiple comparison test:<br>1. Male vs. Female:<br>DA: p=0.251<br>5-HT: **p=0.003<br>NE: p=0.631<br><br>2. Among neuromodulator types:<br>DA vs. 5-HT: male, ***p<0.001; female, p=0.434<br>DA vs. NE: male, ***p<0.001; female, **p=0.006<br>5-HT vs. NE: male, p>0.999; female, p=0.179

2F	DA:<br>N = 14 male;<br>N = 13 female	Two-way repeated-measures ANOVA with Bonferroni post-hoc correction.<br>Column factor: sexes (male vs female)<br>Row factor: across days/conditions	Interaction: F (6, 150) = 3.22, **p=0.005<br>Days/conditions: F (6, 150) = 4.54, ***p<0.001<br>Sexes: F (1, 25) = 5.07, *p=0.033<br>Bonferroni's multiple comparison test:<br><br>1. Across days/conditions (only focus on the following pairs):<br>Cue alone vs. Train day 1: male, ***p<0.001; female, p>0.999<br>Train day 1 vs. Train day 2: male, p>0.999; female, p>0.999<br>Train day 1 vs. Train day 3: male, p=0.189; female, p>0.999<br>Train day 1 vs. Train day 4: male, ***p<0.001; female, p>0.999<br>Train day 2 vs. Train day 3: male, p>0.999; female, p>0.999<br>Train day 2 vs. Train day 4: male, *p=0.012; female, p>0.999<br>Train day 3 vs. Train day 4: male, p>0.999; female, p>0.999<br>Train day 1 vs. Ext. day 1: male, *p=0.011; female, p>0.999<br>Train day 2 vs. Ext. day 1: male, p=0.213; female, p>0.999<br>Train day 3 vs. Ext. day 1: male, p>0.999; female, p>0.999<br>Train day 4 vs. Ext. day 1: male, p>0.999; female, p>0.999<br>Train day 1 vs. Ext. day 2: male, p>0.999; female, p>0.999<br>Train day 2 vs. Ext. day 2: male, p>0.999; female, p>0.999<br>Train day 3 vs. Ext. day 2: male, p>0.999; female, p>0.999<br>Train day 4 vs. Ext. day 2: male, **p=0.004; female, p>0.999<br>Ext. day 1 vs. Ext. day 2: male, p=0.093; female, p>0.999<br><br>2. Male vs. Female:<br>Cue alone: ***p<0.001<br>Train day 1: p=0.690<br>Train day 2: p=0.785<br>Train day 3: p=0.187<br>Train day 4: **p=0.002<br>Ext. day 1: p=0.093<br>Ext. day 2: p=0.840
2G	5-HT:<br>N = 23 male;<br>N = 18 female	Two-way repeated-measures ANOVA with Bonferroni post-hoc correction.<br>Column factor: sexes (male vs female)<br>Row factor: across days/conditions	Interaction: F (6, 234) = 1.51, *p=0.017<br>Days/conditions: F (6, 234) = 4.03, ***p<0.001<br>Sexes: F (1, 39) = 18.9, ***p<0.001<br>Bonferroni's multiple comparison test:<br><br>1. Across days/conditions (only focus on the following pairs):<br>Cue alone vs. Train day 1: male, p=0.610; female, p=0.755<br>Train day 1 vs. Train day 2: male, p>0.999; female, p>0.999<br>Train day 1 vs. Train day 3: male, p>0.999; female, p>0.999<br>Train day 1 vs. Train day 4: male, p>0.999; female, p>0.999<br>Train day 2 vs. Train day 3: male, p>0.999; female, p>0.999<br>Train day 2 vs. Train day 4: male, p>0.999; female, p>0.999<br>Train day 3 vs. Train day 4: male, p>0.999; female, p>0.999<br>Train day 1 vs. Ext. day 1: male, p>0.999; female, p=0.827<br>Train day 2 vs. Ext. day 1: male, p>0.999; female, p=0.514<br>Train day 3 vs. Ext. day 1: male, p>0.999; female, p=0.319<br>Train day 4 vs. Ext. day 1: male, p>0.999; female, p>0.999<br>Train day 1 vs. Ext. day 2: male, p>0.999; female, p=0.090<br>Train day 2 vs. Ext. day 2: male, p>0.999; female, *p=0.049<br>Train day 3 vs. Ext. day 2: male, p>0.999; female, *p=0.027<br>Train day 4 vs. Ext. day 2: male, p=0.948; female, p>0.999<br>Ext. day 1 vs. Ext. day 2: male, p>0.999; female, p>0.999<br><br>2. Male vs. Female:<br>Cue alone: **p=0.005<br>Train day 1: **p=0.003<br>Train day 2: *** p<0.001<br>Train day 3: *** p<0.001<br>Train day 4: p=0.208<br>Ext. day 1: p=0.092<br>Ext. day 2: p=0.285
2H	Ach:<br>N = 11 male;<br>N = 12 female	Two-way repeated-measures ANOVA with Bonferroni post-hoc correction.<br>Column factor: sexes (male vs female)<br>Row factor: across days/conditions	Interaction: F (6, 126) = 1.11, p=0.363<br>Days/conditions: F (6, 126) = 1.40, p=0.219<br>Sexes: F (1, 21) = 0.248, p=0.624
2I	NE:<br>N = 28 male;<br>N = 17 female	Two-way repeated-measures ANOVA with Bonferroni post-hoc correction.<br>Column factor: sexes (male vs female)<br>Row factor: across days/conditions	Interaction: F (6, 258) = 3.26, *p=0.004<br>Days/conditions: F (6, 258) = 1.87, p=0.086<br>Sexes: F (1, 43) = 12.8, ***p<0.001<br>Bonferroni's multiple comparison test:<br><br>1. Across days/conditions (only focus on the following pairs):<br>Cue alone vs. Train day 1: male, p>0.999; female, p>0.999<br>Train day 1 vs. Train day 2: male, p>0.999; female, p>0.999<br>Train day 1 vs. Train day 3: male, p>0.999; female, p>0.999<br>Train day 1 vs. Train day 4: male, p>0.999; female, p>0.999<br>Train day 2 vs. Train day 3: male, p>0.999; female, p>0.999<br>Train day 2 vs. Train day 4: male, p>0.999; female, p>0.999<br>Train day 3 vs. Train day 4: male, p>0.999; female, p>0.999<br>Train day 1 vs. Ext. day 1: male, **p=0.002; female, p>0.999<br>Train day 2 vs. Ext. day 1: male, *p=0.031; female, p>0.999<br>Train day 3 vs. Ext. day 1: male, **p=0.001; female, p>0.999<br>Train day 4 vs. Ext. day 1: male, p>0.999; female, p>0.999<br>Train day 1 vs. Ext. day 2: male, p>0.999; female, p>0.999<br>Train day 2 vs. Ext. day 2: male, p>0.999; female, p=0.602<br>Train day 3 vs. Ext. day 2: male, p>0.999; female, p>0.999<br>Train day 4 vs. Ext. day 2: male, p=0.948; female, p>0.999<br>Ext. day 1 vs. Ext. day 2: male, p>0.999; female, p>0.999

			2. Male vs. Female:<br>Cue alone: p=0.435<br>Train day 1: *p=0.040<br>Train day 2: ** p=0.002<br>Train day 3: p=0.280<br>Train day 4: **p=0.001<br>Ext. day 1: ***p<0.001<br>Ext. day 2: p=0.109
2J	DA:<br>N = 14 male;<br>N = 13 female	Two-way repeated-measures ANOVA with Bonferroni post-hoc correction.<br>Column factor: sexes (male vs female)<br>Row factor: across days/conditions	Days/conditions: F (6, 150) = 28.3, ***p<0.001<br>Sexes: F (1, 25) = 28.3, p=0.126<br>Bonferroni's multiple comparison test:<br><br>Across days/conditions (only focusing on Train days1-4):<br>Train day 1 vs. Train day 2: male, p>0.999; female, p>0.999<br>Train day 1 vs. Train day 3: male, p>0.999; female, p>0.999<br>Train day 1 vs. Train day 4: male, p>0.999; female, p>0.999<br>Train day 2 vs. Train day 3: male, p>0.999; female, p>0.999<br>Train day 2 vs. Train day 4: male, p>0.999; female, p>0.999<br>Train day 3 vs. Train day 4: male, p>0.999; female, p>0.999
2K	5-HT:<br>N = 23 male;<br>N = 18 female	Two-way repeated-measures ANOVA with Bonferroni post-hoc correction.<br>Column factor: sexes (male vs female)<br>Row factor: across days/conditions	Days/conditions: F (6, 234) = 24.3, ***p<0.001<br>Sexes: F (1, 39) = 28.3, ***p<0.001<br>Bonferroni's multiple comparison test:<br><br>Across days/conditions (only focusing on Train days1-4):<br>Train day 1 vs. Train day 2: male, p=0.209; female, p>0.999<br>Train day 1 vs. Train day 3: male, p=0.984; female, p>0.999<br>Train day 1 vs. Train day 4: male, p>0.999; female, p>0.999<br>Train day 2 vs. Train day 3: male, p>0.999; female, p>0.999<br>Train day 2 vs. Train day 4: male, p>0.999; female, p>0.999<br>Train day 3 vs. Train day 4: male, p>0.999; female, p>0.999<br><br>Male vs. Female:<br>Cue alone: p=0.776<br>Train day 1: **p=0.002<br>Train day 2: *** p<0.001<br>Train day 3: *** p<0.001<br>Train day 4: *** p<0.001<br>Ext. day 1: *p=0.025<br>Ext. day 2: p=0.937
2L	Ach:<br>N = 11 male;<br>N = 12 female	Two-way repeated-measures ANOVA with Bonferroni post-hoc correction.<br>Column factor: sexes (male vs female)<br>Row factor: across days/conditions	Days/conditions: F (6, 126) = 0.796, p=0.575<br>Sexes: F (1, 21) = 0.0247, p=0.877
2M	NE:<br>N = 28 male;<br>N = 17 female	Two-way repeated-measures ANOVA with Bonferroni post-hoc correction.<br>Column factor: sexes (male vs female)<br>Row factor: across days/conditions	Days/conditions: F (6, 258) = 44.0, ***p<0.001<br>Sexes: F (1, 43) = 9.82, ** p=0.003<br>Bonferroni's multiple comparison test:<br><br>Across days/conditions (only focusing on Train days1-4):<br>Train day 1 vs. Train day 2: male, p=0.312; female, p>0.999<br>Train day 1 vs. Train day 3: male, p>0.999; female, p=0.056<br>Train day 1 vs. Train day 4: male, ***p<0.001; female, p=0.087<br>Train day 2 vs. Train day 3: male, p>0.999; female, *p>0.043<br>Train day 2 vs. Train day 4: male, p=0.605; female, p=0.067<br>Train day 3 vs. Train day 4: male, p=0.112; female, p>0.999<br><br>Male vs. Female:<br>Cue alone: p=0.487<br>Train day 1: **p=0.005<br>Train day 2: ** p<0.001<br>Train day 3: p=0.170<br>Train day 4: ***p<0.001<br>Ext. day 1: p=0.071<br>Ext. day 2: p=0.818
3J	AUC analysis:<br>N = 8 thirsty and 5 sated males	Two-way repeated-measures ANOVA with Bonferroni post-hoc correction.<br>Column factor: internal states (thirsty vs sated)<br>Row factor: across days	Internal states: F (1, 11) = 7.84, *p=0.02<br>Bonferroni's multiple comparison test (only focusing on comparison between thirsty and sated mice):<br><br>Thirsty vs sated:<br>Re-learning day 1: p=0.84<br>Re-learning day 2: *p=0.01<br>Re-learning day 3: p=0.08
3K	AUC analysis:<br>N= 12 thirsty and 11 sated males	Two-way repeated-measures ANOVA with Bonferroni post-hoc correction.<br>Column factor: internal states (thirsty vs sated)<br>Row factor: across days	Internal states: F (1, 21) = 2.24, p=0.15
3L	AUC analysis:<br>N = 6 thirsty and 5 sated males	Two-way repeated-measures ANOVA with Bonferroni post-hoc correction.<br>Column factor: internal states (thirsty vs sated)<br>Row factor: across days	Internal states: F (1, 9) = 0.913, p=0.36

3M	AUC analysis:<br>N = 15 thirsty and 13 sated males	Two-way repeated-measures ANOVA with Bonferroni post-hoc correction.<br>Column factor: internal states (thirsty vs sated)<br>Row factor: across days	Internal states: F (1, 26) = 4.88, *p=0.04<br>Bonferroni's multiple comparison test (only focusing on comparison between thirsty and sated mice):<br><br>Thirsty vs sated:<br>Re-learning day 1: p=0.57<br>Re-learning day 2: p=0.29<br>Re-learning day 3: p=0.06
3N	AUC analysis:<br>N = 7 thirsty and 6 sated females	Two-way repeated-measures ANOVA with Bonferroni post-hoc correction.<br>Column factor: internal states (thirsty vs sated)<br>Row factor: across days	Internal states: F (1, 11) = 0.954, p=0.350
3O	AUC analysis:<br>N= 10 thirsty and 8 sated females	Two-way repeated-measures ANOVA with Bonferroni post-hoc correction.<br>Column factor: internal states (thirsty vs sated)<br>Row factor: across days	Internal states: F (1, 16) = 20.6, ***p<0.001<br>Bonferroni's multiple comparison test (only focusing on comparison between thirsty and sated mice):<br><br>Thirsty vs sated:<br>Re-learning day 1: p=0.177<br>Re-learning day 2: ***p<0.001<br>Re-learning day 3: p=0.06
3P	AUC analysis:<br>N = 6 thirsty and 6 sated females	Two-way repeated-measures ANOVA with Bonferroni post-hoc correction.<br>Column factor: internal states (thirsty vs sated)<br>Row factor: across days/conditions	Internal states: F (1, 10) = 0.175, p=0.685
3Q	AUC analysis:<br>N = 9 thirsty and 8 sated females	Two-way repeated-measures ANOVA with Bonferroni post-hoc correction.<br>Column factor: internal states (thirsty vs sated)<br>Row factor: across days	Internal states: F (1, 15) = 31.0, ***p<0.001<br>Bonferroni's multiple comparison test (only focusing on comparison between thirsty and sated mice):<br><br>Thirsty vs sated:<br>Re-learning day 1: *p=0.028<br>Re-learning day 2: ***p<0.001<br>Re-learning day 3: ***p<0.001
3R	Peak analysis:<br>N = 8 thirsty and 5 sated males	Two-way repeated-measures ANOVA with Bonferroni post-hoc correction.<br>Column factor: internal states (thirsty vs sated)<br>Row factor: across days	Internal states: F (1, 11) = 4.93, *p=0.048<br>Bonferroni's multiple comparison test (only focusing on comparison between thirsty and sated mice):<br><br>Thirsty vs sated:<br>Re-learning day 1: p=0.513<br>Re-learning day 2: p=0.150<br>Re-learning day 3: *p=0.023
3S	Peak analysis:<br>N = 12 thirsty and 11 sated males;	Two-way repeated-measures ANOVA with Bonferroni post-hoc correction.<br>Column factor: internal states (thirsty vs sated)<br>Row factor: across days	Internal states: F (1, 21) =11.8, **p=0.003<br>Bonferroni's multiple comparison test (only focusing on comparison between thirsty and sated mice):<br><br>Thirsty vs sated:<br>Re-learning day 1: p=0.652<br>Re-learning day 2: p=0.154<br>Re-learning day 3: ***p<0.001
3T	Peak analysis:<br>N = 6 thirsty and 5 sated males	Two-way repeated-measures ANOVA with Bonferroni post-hoc correction.<br>Column factor: internal states (thirsty vs sated)<br>Row factor: across days	Internal states: F (1, 9) =0.00946, p=0.925
3U	Peak analysis:<br>N = 15 thirsty and 13 sated males;	Two-way repeated-measures ANOVA with Bonferroni post-hoc correction.<br>Column factor: internal states (thirsty vs sated)<br>Row factor: across days	Internal states: F (1, 26) = 5.65, *p=0.025<br>Bonferroni's multiple comparison test (only focusing on comparison between thirsty and sated mice):<br><br>Thirsty vs sated:<br>Re-learning day 1: *p=0.036<br>Re-learning day 2: p=0.255<br>Re-learning day 3: p=0.116
3V	Peak analysis:<br>N = 7 thirsty and 6 sated females	Two-way repeated-measures ANOVA with Bonferroni post-hoc correction.<br>Column factor: internal states (thirsty vs sated)<br>Row factor: across days	Internal states: F (1, 11) = 4.23, *p=0.046<br>Bonferroni's multiple comparison test (only focusing on comparison between thirsty and sated mice):<br><br>Thirsty vs sated:<br>Re-learning day 1: p>0.999<br>Re-learning day 2: p=0.224<br>Re-learning day 3: *p=0.023
3W	Peak analysis:<br>N = 10 thirsty and 8 sated females	Two-way repeated-measures ANOVA with Bonferroni post-hoc correction.<br>Column factor: internal states (thirsty vs sated)<br>Row factor: across days	Internal states: F (1, 16) = 16.1, ***p<0.001<br>Bonferroni's multiple comparison test (only focusing on comparison between thirsty and sated mice):<br><br>Thirsty vs sated:<br>Re-learning day 1: p=0.188<br>Re-learning day 2: ***p<0.001<br>Re-learning day 3: ***p<0.001
3X	Peak analysis:<br>N = 6 thirsty and 6 sated females	Two-way repeated-measures ANOVA with Bonferroni post-hoc correction.<br>Column factor: internal states (thirsty vs sated)<br>Row factor: across days	Internal states: F (1, 10) = 0.280, p=0.608
3Y	Peak analysis:<br>N = 9 thirsty and 8 sated females	Two-way repeated-measures ANOVA with Bonferroni post-hoc correction.<br>Column factor: internal states (thirsty vs sated)<br>Row factor: across days	Internal states: F (1, 15) = 37.0, ***p<0.001<br>Bonferroni's multiple comparison test (only focusing on comparison between thirsty and sated mice):<br><br>Thirsty vs sated:<br>Re-learning day 1: ***p<0.001<br>Re-learning day 2: ***p<0.001<br>Re-learning day 3: ***p<0.001

4F	DA:<br>N = 9 males	Two-way repeated-measures ANOVA with Bonferroni post-hoc correction.<br>Column factor: cues (cue 1 vs cue 2)<br>Row factor: across days	Interactions: F (5, 40) = 6.17, ***p<0.001<br>Bonferroni's multiple comparison test (only focusing on comparison between cue 1 and cue 2):<br><br>Cue 1 vs cue 2:<br>CD day 1: p>0.999<br>CD day 2: p=0.143<br>CD day 3: **p=0.005<br>RL day 1: p=0.065<br>RL day 2: p>0.999<br>RL day 3: *p=0.016
4G	5-HT:<br>N = 9 males	Two-way repeated-measures ANOVA with Bonferroni post-hoc correction.<br>Column factor: cues (cue 1 vs cue 2)<br>Row factor: across days	Interactions: F (5, 40) = 7.95, ***p<0.001<br>Bonferroni's multiple comparison test (only focusing on comparison between cue 1 and cue 2):<br><br>Cue 1 vs cue 2:<br>CD day 1: p>0.999<br>CD day 2: p=0.612<br>CD day 3: *p=0.021<br>RL day 1: p>0.999<br>RL day 2: p>0.687<br>RL day 3: ***p<0.001
4H	Ach:<br>N = 7 males	Two-way repeated-measures ANOVA with Bonferroni post-hoc correction.<br>Column factor: cues (cue 1 vs cue 2)<br>Row factor: across days	Interactions: F (5, 30) = 0.700, p=0.628<br>Bonferroni's multiple comparison test (only focusing on comparison between cue 1 and cue 2):<br><br>Cue 1 vs cue 2:<br>CD day 1: p>0.999<br>CD day 2: p>0.999<br>CD day 3: p>0.999<br>RL day 1: p>0.999<br>RL day 2: p>0.999<br>RL day 3: p>0.999
4I	NE:<br>N = 7 males	Two-way repeated-measures ANOVA with Bonferroni post-hoc correction.<br>Column factor: cues (cue 1 vs cue 2)<br>Row factor: across days	Interactions: F (5, 30) = 15.0, ***p<0.001<br>Bonferroni's multiple comparison test (only focusing on comparison between cue 1 and cue 2):<br><br>Cue 1 vs cue 2:<br>CD day 1: p>0.999<br>CD day 2: *p=0.014<br>CD day 3: ***p<0.001<br>RL day 1: p=0.376<br>RL day 2: p=0.395<br>RL day 3: ***p<0.001
4J	DA:<br>N = 13 females	Two-way repeated-measures ANOVA with Bonferroni post-hoc correction.<br>Column factor: cues (cue 1 vs cue 2)<br>Row factor: across days	Interactions: F (5, 60) = 8.99, ***p<0.001<br>Bonferroni's multiple comparison test (only focusing on comparison between cue 1 and cue 2):<br><br>Cue 1 vs cue 2:<br>CD day 1: p>0.999<br>CD day 2: p=0.258<br>CD day 3: ***p<0.001<br>RL day 1: p=0.101<br>RL day 2: p>0.999<br>RL day 3: ***p<0.001
4K	5-HT:<br>N = 7 females	Two-way repeated-measures ANOVA with Bonferroni post-hoc correction.<br>Column factor: cues (cue 1 vs cue 2)<br>Row factor: across days	Interactions: F (5, 30) = 5.70, ***p<0.001<br>Bonferroni's multiple comparison test (only focusing on comparison between cue 1 and cue 2):<br><br>Cue 1 vs cue 2:<br>CD day 1: p>0.999<br>CD day 2: p>0.999<br>CD day 3: p=0.055<br>RL day 1: p=0.063<br>RL day 2: p>0.999<br>RL day 3: *p=0.017
4L	Ach:<br>N = 8 females	Two-way repeated-measures ANOVA with Bonferroni post-hoc correction.<br>Column factor: cues (cue 1 vs cue 2)<br>Row factor: across days	Interactions: F (5, 35) = 1.91, p=0.118<br>Bonferroni's multiple comparison test (only focusing on comparison between cue 1 and cue 2):<br><br>Cue 1 vs cue 2:<br>CD day 1: p=0.456<br>CD day 2: p>0.999<br>CD day 3: p>0.999<br>RL day 1: p>0.999<br>RL day 2: p=0.767<br>RL day 3: p=0.988
4M	NE:<br>N = 8 females	Two-way repeated-measures ANOVA with Bonferroni post-hoc correction.<br>Column factor: cues (cue 1 vs cue 2)<br>Row factor: across days	Interactions: F (5, 35) = 15.4, ***p<0.001<br>Bonferroni's multiple comparison test (only focusing on comparison between cue 1 and cue 2):<br><br>Cue 1 vs cue 2:<br>CD day 1: p>0.999<br>CD day 2: ***p<0.001<br>CD day 3: ***p<0.001<br>RL day 1: *p=0.003<br>RL day 2: p>0.999<br>RL day 3: p>0.999
